# Usefulness and pitfalls of neuroendoscopic tumor biopsy for intracranial malignant lymphoma

**DOI:** 10.1007/s10143-025-03636-5

**Published:** 2025-05-29

**Authors:** Shinjiro Fukami, Kenta Nagai, Nobuyuki Nakajima, Sho Onodera, Jiro Akimoto, Michihiro Kohno

**Affiliations:** 1 Department of Neurosurgery, Tokyo Medical University, 6-7-1 Nishishinjuku, Shinjuku-ku, Tokyo, 160-023 Japan; 2 Department of Neurosurgery, Kohsei Chuo General Hospital, Tokyo, Japan

**Keywords:** Malignant lymphoma, Biopsy, Neuroendoscope, Complication

## Abstract

**Purpose:**

Neuroendoscopic biopsy has various advantages, such as being able to collect larger tissue samples and achieving more efficient hemostasis compared to needle biopsy. The purpose of this study is to review the surgical techniques, accuracy of pathological diagnosis, and perioperative complications of patients with intracerebral malignant lymphoma who were diagnosed mainly by neuroendoscopic biopsy.

**Methods:**

A total of 65 patients diagnosed as having malignant lymphoma, via mainly neuroendoscopic biopsy, were analyzed retrospectively. A flexible neuroendoscope was used for ventricular and paraventricular lesions, which was inserted via the lateral ventricle. For intraparenchymal lesions, rigid scopes with navigation systems were used.

**Results:**

The main location of the tumors was in the intraventricular/paraventricular regions in 22 patients, in the deep white matter in 22 patients, in the basal ganglia in 14 patients, and in the cerebellum in 5 patients. Two patients had a lymphomatosis cerebri-like lesion. Lymphoma was diagnosed in 58 of the 65 patients, with most patients diagnosed as having diffuse large B-cell lymphoma. Incorrect diagnoses were owing to inappropriate samples, sentinel lesions, or preoperative treatments, such as with steroids. Complications included small cerebral hematoma in 5 patients, severe cerebral edema in 3 patients (2 fatal), and brain abscess and arterial bleeding leading to infarction in 1 case each.

**Conclusion:**

Neuroendoscopic biopsy is a reliable diagnostic approach for intracranial malignant lymphomas, particularly those in deep or intraventricular/paraventricular locations. However, caution is particularly required for patients with severe cerebral swelling, which may cause death, and those with prior sentinel lesions.

## Introduction

Intracerebral malignant lymphomas are often found in the paraventricular region of the brain, including the deep white matter bordering the ventricular wall [1, 2]. The main treatment methods are chemotherapy and radiation therapy, and in most cases, removal of the tumor is not required. Therefore, a biopsy is often performed to make a pathological diagnosis. Stereotactic needle biopsy is the most commonly used biopsy technique for deep lesions, but the risk of intracerebral hematoma is not negligible [3–9], and we have been actively using neuroendoscopic biopsy, which enables more efficient hemostasis and collection of a larger sample volume. We have also reported the usefulness of neuroendoscopic surgery for paraventricular gliomas [10]. For lesions close to the surface of the brain, open biopsy can be performed. Intracranial lymphomas, on the other hand, are often periventricular and located deep in the brain, and rarely occur within the ventricles, and hence appear to be a good indication for endoscopic biopsy [11]. Intracerebral malignant lymphomas often occur in older patients and in patients with low Karnofsky Performance Status, and we believe that neuroendoscopic biopsy, which is a minimally invasive procedure, is a preferable approach. In this study, we reviewed the imaging characteristics, surgical techniques used, accuracy of pathological diagnosis, and perioperative complications of patients with intracerebral malignant lymphoma who were diagnosed mainly by neuroendoscopic biopsy performed at our institution.

## Materials and methods

### Patients and study design

The subjects were a total of 65 patients (median age: 69 years), ranging from 43- to 84-years old, who were pathologically diagnosed as having intracerebral malignant lymphoma by mainly endoscopic biopsy, neuroradiological imaging, cerebrospinal fluid (CSF) cytology, and/or clinical course for about 18 years since September 2007 at Tokyo Medical University Hospital, Tokyo Medical University Ibaraki Medical Center, and Tokyo Medical University Hachioji Medical Center. The series is slightly dominated by older men, with 36 men and 29 women. The data includes not only patients in whom lymphoma was confirmed by neuroendoscopic biopsy, but also patients in whom lymphoma was detected by intraocular or CSF cytology, even if it was not confirmed by neuroendoscopic biopsy. This study was designed to clarify the diagnostic and complication rates of biopsy procedures and the details of the complications through a retrospective, single-arm analysis of all eligible patients who underwent surgery performed by 2 surgeons (S.F. or N.N.). This study was conducted in accordance with the Helsinki Declaration, and was approved by the Ethical Review Board of Tokyo Medical University Hospital (study approval no.: T2020-0244).

### Biopsy procedure

**Fig. 1:**
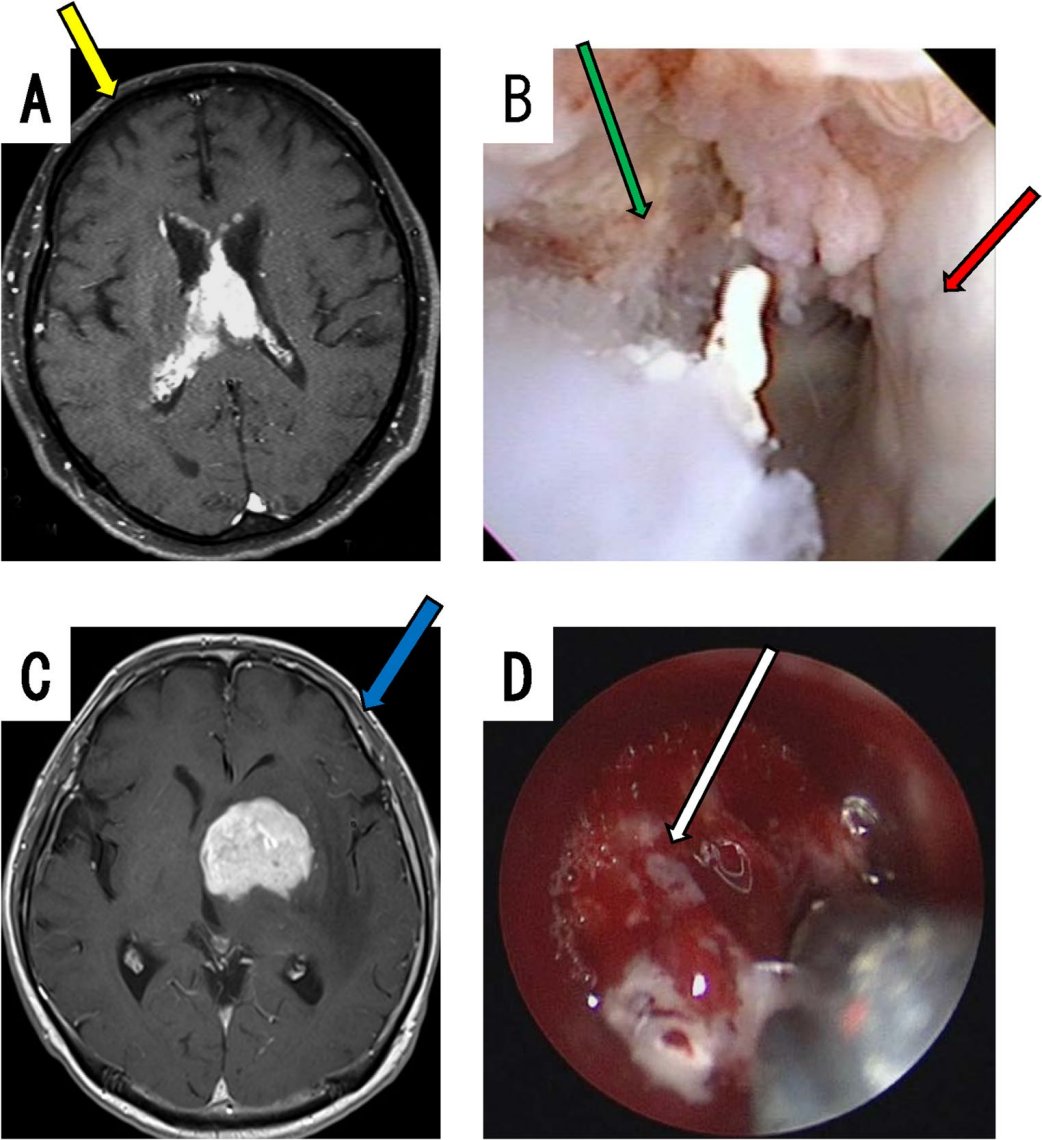
Two representative cases of neuroendoscopic biopsy. Case A: MRI displayed enhanced lesions in the septum pellucidum within the ventricles, and the lateral ventricular walls were also partly contrast-enhanced (**A**). The lesion was approached from the right anterior horn of the lateral ventricle (**A**, yellow arrow: direction of sheath insertion). An obvious lesion was found within the ventricle (**B**, green arrow), and the ventricular wall were reduced in transparency (**B**, red arrow). Case B: MRI displayed a lesion in the left caudate head (**C**). The lesion was approached using a ridged scope (**C**, blue arrow: direction of sheath insertion). Endoscopy demonstrated that the tumor was dark red (**D**, white arrow)

Biopsy for pathological diagnosis in all patients was performed under general anesthesia. For intraventricular and paraventricular lesions (representative case, Fig. 1A), the lesion was approached from the anterior horn of the lateral ventricle on the tumor side, using a flexible endoscope (Fig. 1A, yellow arrow). When the lesion was inside of the third ventricle, the right side of the lateral ventricle was used. Flexible scopes, namely, either a videoscope (VEF TYPE V; OLYMPUS, Tokyo, Japan) or a fiberscope (NEU-4 L; Machida Endoscope Co., Chiba, Japan) with a transparent sheath (Neurosheath, 17.5 French gauge; Medikit, Tokyo, Japan) were used. If a tumor is not evident when using a videoscope, narrow-band imaging (NBI) can sometimes be used to identify the tumor [12]. Lesion sampling was performed using flexible forceps (Machida Endoscope Co., Chiba, Japan). For hemostasis, sufficient irrigation with Ringer’s lactate or artificial CSF (ARTCEREB; Otsuka Pharmaceutical Factory, Inc., Tokushima, Japan) was routinely performed. In patients in whom conventional hemostasis was difficult, electric coagulation was used (PAL-1, not available for sale in 2025, Japan Medical Dynamic Marketing, Inc., Tokyo, Japan or MICROTAZE, Alfresa, Tokyo, Japan). If the patient had hydrocephalus, third ventriculostomy or septostomy using balloon expansion was also performed (Fogarty, Edwards Lifesciences Corp., CA, USA or Expanser balloon catheter, Fuji Systems Co., Tokyo, Japan). For lesions distant from the ventricles, such as the basal ganglia (representative case, Fig. 1C), biopsy was performed using a rigid scope (EndoArm 2.7 or 4 mm, OLYMPUS, Tokyo, Japan or Skull Base Endoscope 2.7 mm, Karl Storz, Tuttlingen, Germany). The transparent sheath (NEUROPORT, OLYMPUS, Tokyo, Japan) was guided directly into the lesion under brain navigation (Brain Lab, Munich, Germany). The tumor forceps were universal tumor forceps used in sinus surgery. Hemostasis was achieved using a cauterization suction tube with insulation coating on all but the tip (Fujita Medical Instruments, Tokyo, Japan), oxidized cellulose cotton, and/or fibrin glue. Biopsy specimens were analyzed for a pathological diagnosis, mainly using immunohistochemical staining for cluster of differentiation (CD) 20, CD3, and MIB1 (monoclonal antibody against Ki-67 protein), in addition to hematoxylin and eosin (H.E.) staining. Cefazolin sodium was usually used as a perioperative antibiotic from just before surgery until 2 days after surgery, but other antibiotics were used in cases of combined infection or cephem allergy. Intraoperative rapid pathological analysis was performed when possible, but sometimes it could not be performed during emergency surgery or owing to the unavailability of staff from the pathology department. Whether the sampling area was in the correct location or not was confirmed by plain computed tomography (CT) or magnetic resonance imaging (MRI) within 3 days after the surgery.

## Results

### Tumor location and characteristics

A total of 22 patients had masses within the ventricles or in the paraventricular areas, such as the corpus callosum. Two patients had a diffuse lymphomatosis cerebri-like lesion, which has been reported in recent years [13], with extensive high-signal areas in the white matter and several small nodules on the ventricular walls on fluid-attenuated inversion recovery (FLAIR) MRI. A total of 22 patients had a tumor that was predominantly located in the deep cerebral white matter, such as the frontal lobe (12 patients). In 14 patients, the main locus of the tumor was in the basal ganglia, including the caudate nucleus. In 5 patients the tumor was located in the cerebellar hemisphere or the cerebellar peduncle. Eight patients with intraventricular or paraventricular tumors also had hydrocephalus. In cases in which more than 1 lesion was suspected, the largest site was considered as the tumor location. Regarding the immunological background of the patients, there were no patients with human immunodeficiency virus (HIV), 2 on steroids, 1 patient using methotrexate internally for rheumatoid arthritis, and 1 on immunosuppressive drugs after a kidney transplant. These data are presented in Table 1.

**Table 1:** Clinical features of the 65 patients with malignant lymphoma

	Number	Percentage (%)
**Sex**		
Male	36	55.4
Female	29	44.6
**Age** (years)	Median: 69 (43–84)	
< 60	20	30.8
60–74	27	41.5
> 75	18	27.7
**Tumor location**		
Intraventricle / paraventricle	22	33.8
Cerebrum	22	33.8
Frontal	12	
Temporal	2	
Parietal	3	
Occipital	5	
Basal ganglia	14	21.5
Caudate	5	
Thalamus	6	
Other	3	
Cerebellum	5	7.7
Cerebri pattern	2	3.1
**Hydrocephalus**		
Present	8	12.3

### Surgical procedure

**Fig. 2:**
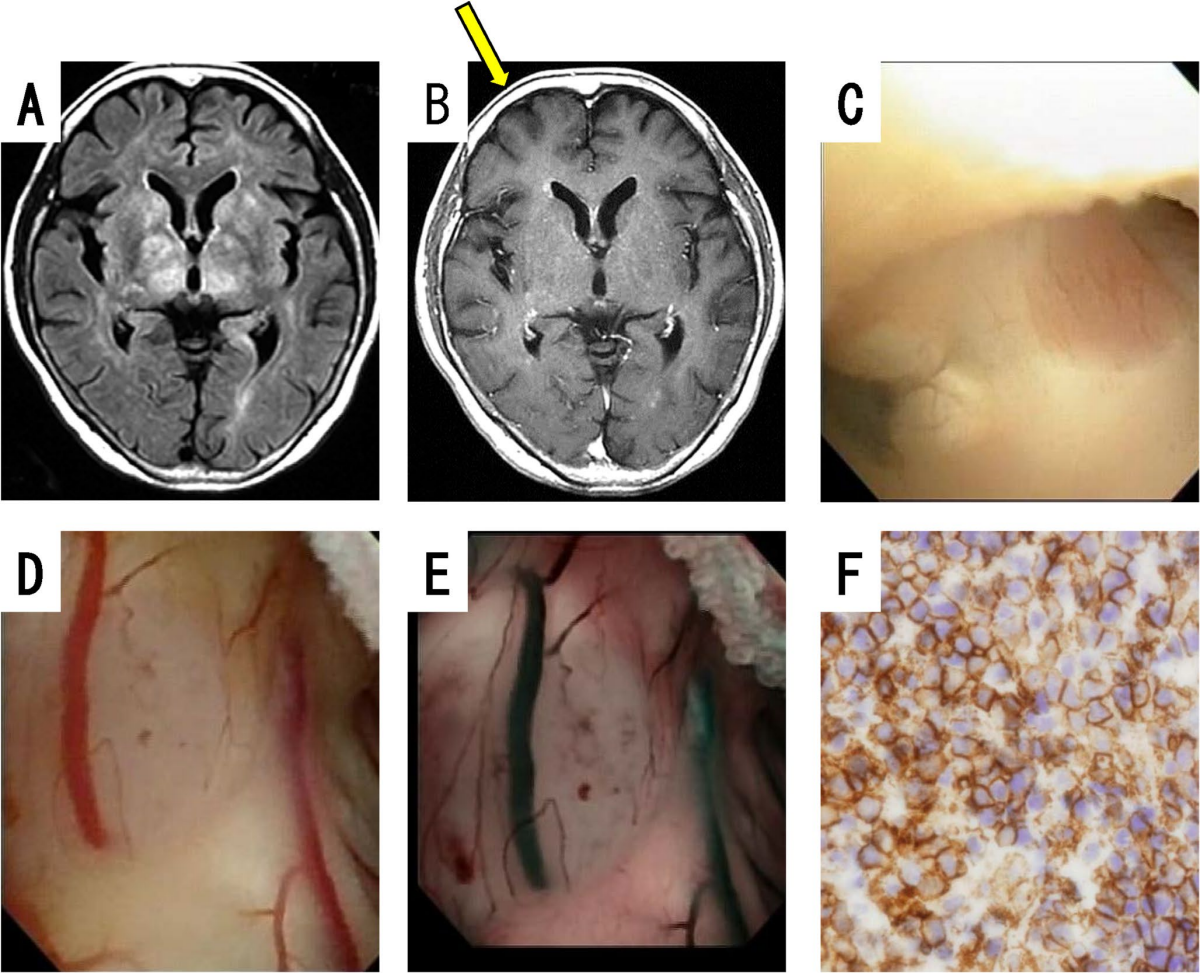
A case of lymphomatosis cerebri. Case C: Brain FLAIR MRI displayed a high signal in the bilateral deep white matter and basal ganglia (**A**), with spotty contrast of the ventricular walls (**B**). Neuroendoscopic biopsy via the anterior horn of the right ventricle (**B**, yellow arrow: direction of sheath insertion). Intraoperative neuroendoscopy displayed decreased transparency of the ventricular walls and multiple nodules, and pink nodules were observed in the posterior third ventricular floor (**C**) and lateral ventricular walls (**D**), which were evident on NBI (**E**). The biopsy specimen consisted of large tumor cells stained with CD20 antibodies (**F**), and was diagnosed as DLBCL

Biopsies using a flexible scope were performed in 28 patients with intraventricular and paraventricular tumors. Biopsy of intraventricular tumors (Fig. 1B) is not difficult because the target is obvious. On the other hand, in patients in whom there was no obvious mass in the ventricles, biopsies of the bulging or nodular areas of the brain surface were performed, occasionally referring to changes in the color of the ventricular wall, although the difficulty is increased. All transventricular wall biopsies were performed using a flexible scope, and the ventricular walls had reduced transparency and/or white spotting patterns, suggesting dissemination of the tumor (Figs. 1B and 2C). Of the 8 patients with hydrocephalus, third ventriculostomy was performed in 2 patients and septostomy in 4 patients at the same time as the biopsy using a flexible scope. Biopsies were performed for 37 intraparenchymal brain tumors using a rigid scope with navigation. The tumor was accurately biopsied after endoscopic confirmation of a dark red tumor (Fig. 1D). In both biopsy procedures, the wound was closed after confirming hemostasis. Because only a small volume of the specimens was collected, they could not be evaluated quantitatively or qualitatively. The biopsy specimens collected using a rigid scope tended to be larger than those collected using a flexible scope, in terms of visual impression. These data are presented in Table 2.

**Table 2:** Methods, pathology, and complications of the biopsy (*n* = 65)

	Number	
**Method of biopsy**		
Flexible scope	28	
With third ventriculostomy		2
With septostomy		4
Solid scope with navigation	37	
**Pathological diagnosis from the biopsy**		
Diagnosed as lymphoma	58	
DLBCL		54
PTLD		1
Lymphoma but subtype not identified		3
Unable to diagnose lymphoma	7	
Inappropriate specimen		3
Sentinel lesions suspected		2
After steroid use		1
Inappropriate route of biopsy		1
**Complication**		
Small hematomas	5	
Severe brain edema	3	
Death within 1 month after surgery		2
Cerebral infarction owing to hemostasis of the perforating artery	1	
Abscess in the path of the sheath	1	

DLBCL, diffuse large B-cell lymphoma; PTLD, post-transplant lymphoproliferative disorder

### Diagnosis and complications

The rate of diagnosis of malignant lymphoma by biopsy was 89% (58/65). In the 7 patients who were not diagnosed by biopsy, no tumor cells could be detected in the biopsy specimens, and the final diagnosis of malignant lymphoma was made after reoperation, CSF cytology, and/or the course of the disease, such as the development of intraocular lesions. Reasons for the unsuccessful diagnosis of 7 patients from the endoscopic biopsies were as follows: in 3 patients (2 intraventricular tumors biopsied using a flexible scope, and 1 thalamic tumor biopsied using a rigid scope) the specimens were inappropriate, such as being too small, 2 patients showed non-neoplastic precursor changes called sentinel lesions, 1 patient underwent biopsy by an incorrect approach route, and in 1 patient the tumor vanished, probably owing to preoperative steroid induction. Of the 58 patients diagnosed as having lymphoma, 54 were diagnosed as having diffuse large B-cell lymphoma (DLBCL), and 1 patient as having post-transplantation lymphoproliferative disorder. On the other hand, 3 patients were diagnosed as having malignant lymphoma but could not be subtyped because of inappropriate specimens, such as a large amount of tissue around the tumor or very small amounts of the tumor. Perioperative complications were as follows: 5 patients had small hematomas in the path of the sheath or within the tumor, 3 patients had severe brain edema, 1 patient had a brain abscess in the tract requiring reoperation for its removal, and 1 patient had arterial bleeding during biopsy of a thalamic tumor, in which intraoperative hemostasis was difficult, and a small perforating branch infarction occurred postoperatively. All 5 patients with hematoma did not require surgery and did not experience permanent symptoms. Of the 3 patients with cerebral swelling, 2 patients died owing to cerebral herniation within a month after the surgery, and 1 patient was successfully treated by hyperosmotic diuretics and steroid pulse therapy. These data are presented in Table 2.

### Representative cases

#### Case A: intraventricle lesion

A 75- to 80-year-old man developed memory disturbance. Gd-enhanced T1-weighted MRI displayed homogenous enhanced lesions in the septum pellucidum within the ventricles, and the lateral ventricular walls were also partly contrast-enhanced (Fig. 1A). The endoscopic approach using a flexible scope was performed from the right anterior horn of the lateral ventricle. The yellow arrow in Fig. 1A indicates the approximate direction of sheath insertion. In the endoscopic view, an obvious lesion was detected within the ventricle (Fig. 1B, green arrow), and the ventricular wall was reduced in transparency (Fig. 1B, red arrow). The pathological finding was DLBCL.

#### Case B: basal ganglia lesion

A 50- to 55-year-old man developed memory disturbance and right hemiparesis. Gd-enhanced T1-weighted MRI displayed homogenous enhanced lesion in the left head of the caudate (Fig. 1C). The lesion was approached using a rigid endoscope. The sheath of the endoscope was inserted directly into the lesion using navigation (Fig. 1C, blue arrow). Endoscopy demonstrated that the tumor was dark red (Fig. 1D, white arrow).

#### Case C: diffuse lymphomatosis cerebri-like lesion

The patient was a 70- to 75-year-old woman. She developed loss of memory, and was suspected of having Alzheimer disease. Brain MRI displayed a high FLAIR signal in the bilateral deep white matter and basal ganglia (Fig. 2A), with spotty contrast of the ventricular walls (Fig. 2B). Therefore, neuroendoscopic biopsy was considered superior to stereotactic surgery because the endoscope can visualize spotty contrast lesions. Neuroendoscopic biopsy with a flexible scope was performed via the anterior horn of the right ventricle (Fig. 2B, yellow arrow). Intraoperative neuroendoscopy displayed decreased transparency of the ventricular walls, and multiple nodules (Fig. 2C). In addition, pink nodules were observed in the posterior third ventricular floor (Fig. 2C) and lateral ventricular walls (Fig. 2D), which were evident on NBI (Fig. 2E). The biopsy specimen consisted of large tumor cells stained with Cluster of Differentiation (CD) 20 antibodies (Fig. 2F), and the pathological diagnosis was DLBCL. After the pathological diagnosis, the patient was treated with high-dose methotrexate therapy, and her cognitive symptoms improved.

#### Case D: suspected sentinel lesion

**Fig. 3:**
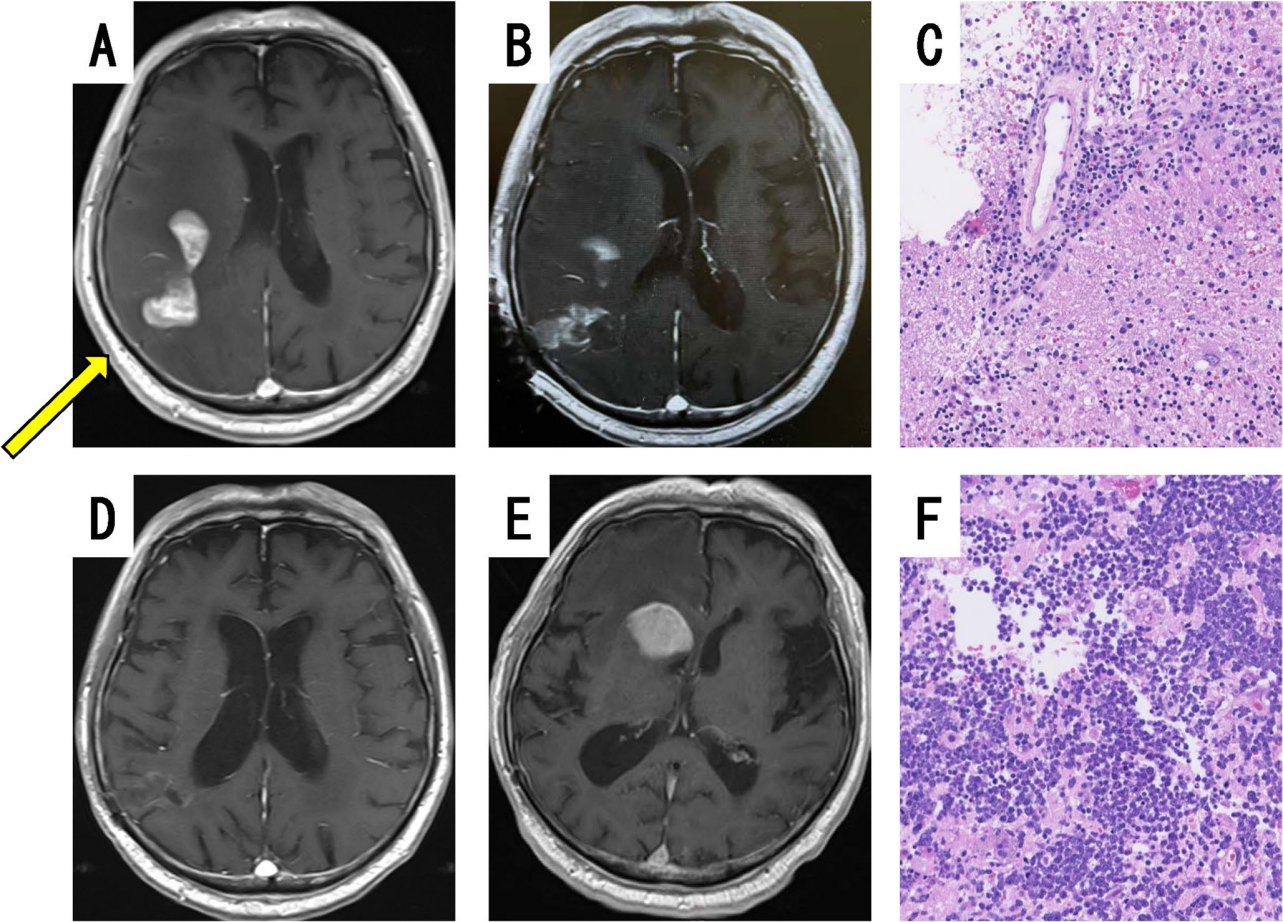
A case of sentinel lesion. Case D: Brain MRI displayed a heterogeneous enhanced mass in the right temporal lobe (**A**, yellow arrow: direction of sheath insertion). A biopsy was performed in the appropriate area, which was confirmed 2 days after the biopsy (**B**). H.E. staining of the neuroendoscopic biopsy specimen revealed nonspecific inflammation and perivascular plasma cell clusters, but no lymphoma cells (**C**). After steroid therapy, the lesion disappeared (**D**). Two months after the steroid therapy, a new homogeneously contrasted mass appeared in the right caudate nucleus (**E**). H.E. staining of the open biopsy specimen revealed densely clustered large monoclonal tumor cells (**F**)

The patient was a 65- to 70-year-old man. He presented with mild left hemiparesis. His initial MRI displayed a heterogeneous enhanced mass in the right temporal lobe (Fig. 3A). The lesion was approached using a rigid endoscope. Contrast-enhanced MRI 2 days after the biopsy procedure demonstrated lesion changes posterior to the right temporal lobe, indicating that the biopsy was performed in the proper area (Fig. 3B). The biopsy specimen showed nonspecific inflammation and perivascular plasma cell clusters, but no lymphoma cells (Fig. 3C). Bacteriological tests were also negative, and the diagnosis was nonspecific inflammatory disease. Therefore, steroid pulse therapy was started, after which the lesions temporarily resolved (Fig. 3D) and the patient’s left paralysis improved. However, 2 months after the start of steroid pulse therapy, his consciousness became impaired, and brain MRI displayed a homogeneously contrast-enhanced mass in the right caudate nucleus (Fig. 3E), which led to an open biopsy being performed aiming to completely remove the contrast-enhanced lesion for a more reliable pathological diagnosis. This second biopsy demonstrated densely clustered large monoclonal tumor cells (Fig. 3F) that were positive for CD20, and a diagnosis of DLBCL was made. Based on the patient’s history, the initial lesion appeared to be a sentinel lesion that subsequently developed into malignant lymphoma.

#### Case E: severe brain swelling after neuroendoscopic biopsy

**Fig. 4:**
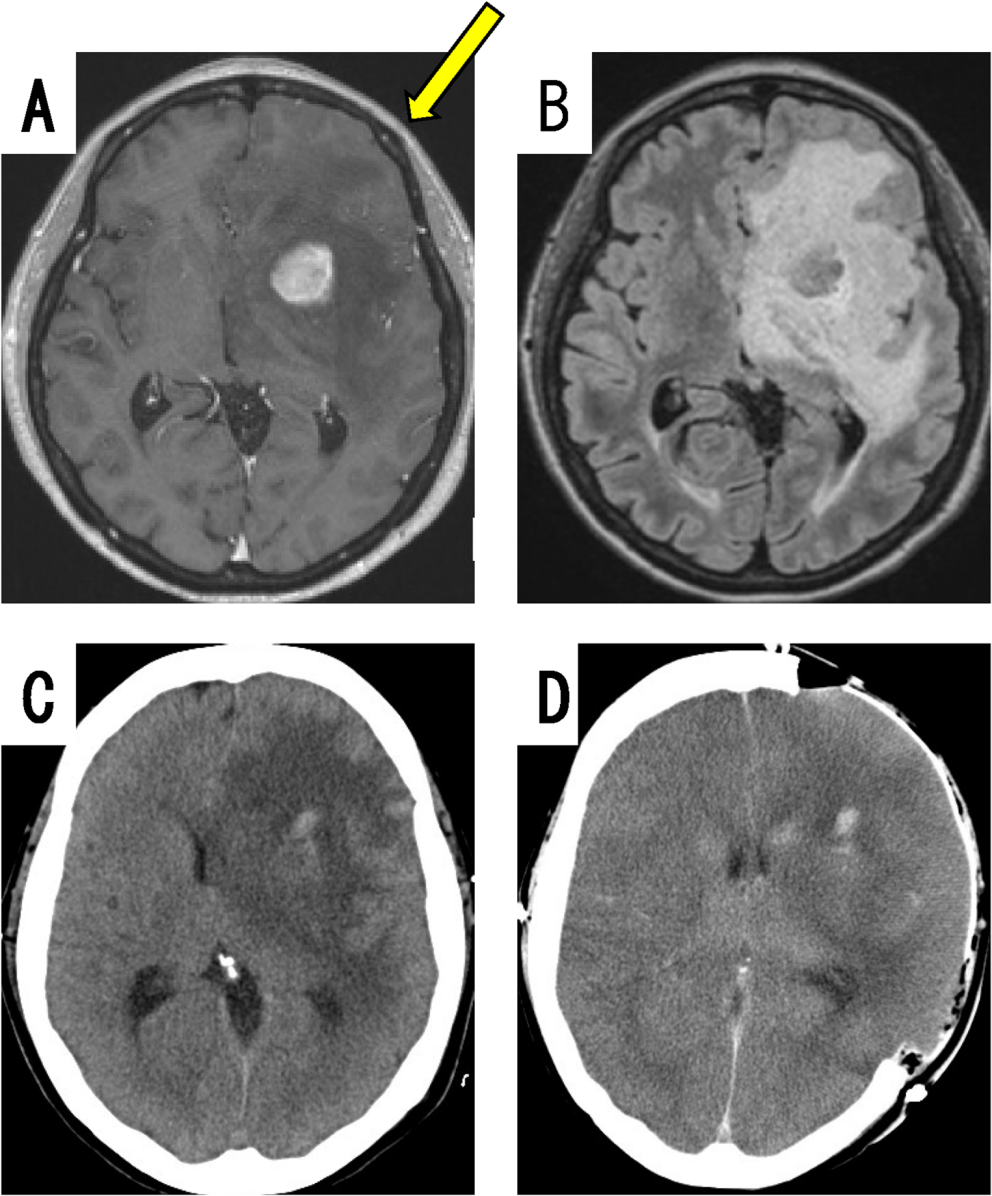
A case of severe brain swelling. Case E: Brain MRI displayed a small enhanced mass in the deep left frontal lobe (**A**, yellow arrow: direction of sheath insertion), together with severe brain edema in FLAIR (**B**). Brain CT 27 hours after the biopsy displayed prominent cerebral edema with midline shift (**C**). Brain CT after external decompression displayed unclear corticomedullary boundaries (**D**), suggesting severe cerebral dysfunction

The patient was a 55- to 60-year-old woman. She presented with right hemiparesis and aphasia. An initial MRI displayed a homogeneous enhanced mass in the deep left frontal lobe (Fig. 4A), with severe brain edema on FLAIR (Fig. 4B). Neuroendoscopic biopsy using a rigid scope was performed via the transcortical approach, because the tumor mass was small compared with the brain edema, and the lesion was deep. Immediately after the surgery, the patient was awake and hence extubated, but intractable convulsions occurred from 26 hours postoperatively. Brain CT performed 27 hours postoperatively displayed prominent cerebral edema with a midline shift (Fig. 4C). Immediate emergency external decompression was performed, but brain CT after the reoperation displayed unclear corticomedullary boundaries (Fig. 4D), suggesting severe cerebral dysfunction. The patient died 20 days after the biopsy.

## Discussion

Intracranial malignant lymphoma is characterized by a periventricular growth pattern, with 30–50% of cases being of the subependymal type, and 40–100% demonstrating tumor extension to the ependymal surface. Because of the deep location of the lesion, stereotactic needle biopsy or neuroendoscopic biopsy is often considered to be more suitable than open biopsy. When the lesions are within or near the ventricles, biopsy via the transventricular route may also be possible. The difficulty of operation increases when there is no obvious mass in the ventricle; however, by close observation of the ventricular wall and with the use of special endoscopic optical techniques, such as NBI, it can be possible to perform a biopsy from inside the ventricle [12]. We use different types of endoscopic biopsy techniques for lymphomas depending on the site of the tumor, using either a flexible or rigid scope. For intraventricular/paraventricular lesions, a flexible scope is preferred, as it enables extensive observation of the ventricular wall and biopsy of multiple sites, as well as septostomy for hydrocephalus at the same time. Disadvantages of the flexible scope include the lack of navigation guidance and that it is more difficult to manipulate and perform hemostasis than a rigid scope. Navigation guidance is used in biopsy areas away from the ventricles, as it is necessary to reach the target precisely. For these sites, we therefore performed biopsy using a rigid scope. For lymphomas in the basal ganglia or deep white matter, stereotactic needle biopsy is also considered. However, intracranial hemorrhage is the most common complication of needle biopsy, occurring in 4.8–9.0% of cases, with a high morbidity rate of 5–6.9% and a mortality rate of 0–2.8% [3–8]. This indicates a substantial risk, as the stereotactic procedure is performed without direct visualization, making it difficult to control bleeding during the operation. On the other hand, neuroendoscopic biopsy appears to be more useful than stereotactic needle biopsy because it can accurately confirm a tumor and adequate hemostasis can be performed, and it also provides a high quality specimen with a large tumor volume. In our department, hemostasis appeared to be easier to perform for lymphomas than for gliomas [10]. In our patient series, we encountered 5 patients with small hematomas in the path of the sheath or within the tumor, and 1 patient with arterial bleeding, which was difficult to control intraoperatively. Nevertheless, both hemorrhagic complications were minor and asymptomatic owing to adequate intraoperative hemostasis. Both empirically and in reports in the literature, malignant gliomas are often hypervascularized, whereas cerebral lymphomas are often avascularized on cerebral angiography [14]. In our experience, endoscopic findings of hemorrhagic arterial vessels appear to be less common in lymphomas than in malignant gliomas. Hemorrhagic complications are frequent in both patients with lymphoma and those with grade 4 glioma who undergo stereotactic biopsy, but complications associated with symptoms and mortality are more frequent in patients with malignant glioma [15, 16].

Positive diagnosis rates for intracranial malignant lymphomas using stereotactic needle biopsies have been reported to be about 90% [1, 17]. According to a Japanese multicenter neuroendoscopic study, 8.7% (62/714) of ventricular and paraventricular neuroendoscopic biopsies were performed for lymphoma and associated disorders [18]. In this previous report, among the patients who were older than 70 years of age, 40.5% had lymphoma [18]. Navigation-guided rigid endoscope biopsy has been reported to be a safe method with a high diagnostic rate (97%), and enables the collection of a large sample within a short operating time [19, 20]. The diagnostic rate in this study was not very high, at 89% (58/65). Of the 7 nondiagnosed cases, all but 2 sentinel lesions could have been diagnosed if there had been no errors in technique or management. The following are some suggestions: avoid steroid use prior to biopsy, use navigation even by the ventricular route, obtain larger specimens, and observe tissue coloration to ensure tumor sampling. In all cases of intraventricular tumors using a flexible scope, there was a decrease in the transparency of the ventricular wall, and in some cases, nodular changes were observed, such as in Case C. Such ventricular wall changes can be seen as inflammatory changes and as tumor dissemination. In the patients in this series, the presence of lymphoma was confirmed by simultaneous biopsy, and therefore, dissemination was highly suspected. Endoscopic biopsy enables observation of the ventricular walls in addition to tissue collection, and in the future, chemotherapy and radiation therapy may be tailor-made for each patient based on intraoperative findings, such as the degree of tumor cell dissemination.

The 2 perioperative deaths and the 1 case of transient loss of consciousness that occurred in our patient series were associated with severe preoperative cerebral edema. Regarding such intraparenchymal tumors causing severe brain edema, there is a risk of brain herniation owing to the worsening of brain swelling immediately after endoscopic biopsy, and it appeared safer to perform resection aimed at internal decompression of the tumor than only biopsy. The usefulness of surgical resection for cerebral lymphoma has also been reported in recent years [21, 22]. From the viewpoint of medical safety, therefore, for tumors with volumes or brain edema large enough to cause a midline shift or impairment of consciousness, it may be desirable to perform internal decompression and/or resection of the tumor, even for deep lesions, instead of neuroendoscopic biopsy.

Regarding the pathological diagnosis, 2 patients in our series had non-neoplastic precursor changes called sentinel lesions. Sentinel lesions are non-neoplastic demyelinating and/or inflammatory changes resulting from an early immune response to the tumor, and often initially present with lymphocyte-dominant and T-cell-dominant pathogenesis [23, 24]. Sentinel lesions show hyperaccumulation on fluorodeoxyglucose- Positron Emission Tomography (FDG-PET), and may appear tumor-like on imaging [25]. Remote recurrence is observed several months after the initial biopsy, and recurrent lesions often lead to the diagnosis of malignant lymphoma. MRI displays a high signal on T2-weighted images and a heterogenous contrast effect on T1-weighted images in many patients [24]. The patient of case D in this series also demonstrated contrast-enhanced lesions with heterogenous contrast, and at a different site at the time of recurrence. If lymphoid tumors are suspected but no malignant lymphoma tissue is detected on initial biopsy, careful postoperative follow-up imaging, for example, contrast-enhanced brain MRI every 2 to 3 months should be performed, and if necessary, a second biopsy should be strongly considered.

A limitation of this study is that it was a single-arm, retrospective study. As the study was performed over a long period (18 years) in 3 institutions, there is the potential of selection bias and heterogeneity in perioperative management. In addition, as there were only 2 primary surgeons, there was high consistency in the technique, but this may have led to bias in the results. In the future, it would be desirable to perform a multicenter, multioperator, prospective comparative study to clarify the usefulness of neuroendoscopic biopsy, particularly to confirm its superiority over stereotactic or open biopsy.

## Conclusions

Neuroendoscopic biopsy is a reliable diagnostic approach for intracranial malignant lymphomas, particularly those in deep or intraventricular/paraventricular locations, by enabling efficient hemostasis, sampling of large tissue specimens, and direct visualization of lesions. In this study, 65 patients who underwent neuroendoscopic biopsy demonstrated a high diagnostic accuracy (89%) and manageable complication rates, with unfavorable outcomes largely associated with pre-existing severe brain edema. This underscores the need for careful patient selection and consideration of surgical resection for decompression in patients with substantial brain edema. There may also be sentinel lesions in which biopsy specimens show no lymphoma cells, in which case frequent follow-up and rebiopsy should be considered.

The utility of neuroendoscopy extends beyond diagnosis, offering intraoperative insights that may provide useful information towards individualized therapy, such as tumor cell dissemination. Future advancements in neuroendoscopic techniques and tailored treatment strategies hold promise towards improving outcomes in patients with intracranial malignant lymphoma.

## Data Availability

No datasets were generated or analysed during the current study.
